# Empowering political participation through artificial intelligence

**DOI:** 10.1093/scipol/scy064

**Published:** 2018-11-05

**Authors:** Paulo Savaget, Tulio Chiarini, Steve Evans

**Affiliations:** 1 University of Cambridge, Cambridge, UK; 2 Strategic Division, National Institute of Technology, Rio de Janeiro, Brazil

**Keywords:** artificial intelligence, political participation, open-data, democracy, public administration

## Abstract

Technologies based on artificial intelligence (AI) can radically change the existing political paradigm, empowering more diffused forms of political participation beyond elections—especially in the emergent worldwide context of unrestricted disclosure of governmental data online. The objective of this research is to investigate how civil society can use AI-based technologies to empower political participation. A sample of 721 publications was conducted through a combination of bibliometric analysis and systematic review, which revealed the characteristics and the nascent state of literature. This was followed by an exploratory Case Study, conducted through in-depth interviews and participant observation and supplemented by secondary materials. The content of the Case Study was extensively and systematically analysed through textual coding. We depicted a framework of how civil society can use AI-based technologies to nurture diffused political participation. This framework scrutinizes six focal areas and their respective dominant traits and descriptive features, aiming at contributing to guiding academic studies and political endeavours.

## Introduction

Non-biological intelligent technologies—so-called Artificial Intelligence (AI)—have proven to be highly pervasive within social, technological and economic systems alike. Thanks to the ever-continuing advances and diffusion of a constellation of interconnected technologies—such as semiconductor chips, transistors, computer processors, memory capacity, the World Wide Web, cloud storage, big data analysis software and sophisticated neural networks—AI has gained momentum to progressively shape socio-technical system change (Schatsky *et al*., 2014; Kelnar, 2016; Schwab, 2016; Huber, 2017; Makridakis, 2017). There has also been a recent worldwide increase in disclosure of public data that can be freely used, compared and shared, thereby improving transparency and scrutiny of government activities and expenditures. AI-related technologies and public open-data, when combined, can thus reshape the existing political paradigm, opening up scope for more diffused forms of political participation, beyond voting in sporadic elections.

Political science recognizes the importance of citizen participation and public engagement to democratic processes (Pateman, 1970; Michels, 2011; Archibugi and Cellini, 2017); however, very little is known about how AI-based technologies and the disclosure of public data can empower civil society. The studies available in the literature which deal with the relationship between political science and AI are mainly focused on technical assistance to decisionmaking procedures and on how AI-based technologies can be used by existing governance structures (Milano *et al*., 2014).

This article addresses the following research question: how can AI-based technologies be used to empower political participation? We employed a multi-method approach, starting with the bibliometric analysis and systematic review of a sample of 721 documents. This was followed by an exploratory Case Study, which is particularly capable of providing unprecedented insights into a rather nascent domain. This Case Study was conducted through in-depth interviews and participant observation and subsequently supplemented by secondary materials. Content was extensively and systematically analysed through textual coding, resulting in a framework on how AI can be used by civil society to empower more diffused forms of political participation.

Beyond this introduction, the article is structured as follows. Section 2 sets up our background literature, briefly presenting the controversies regarding the impact of AI-based technologies on democracy. Section 3 presents the research design of this article, including how we collected, processed and analysed data to address our research questions. This section also discusses the results of our biblio-metric analysis and our literature review and briefly describes the characteristics of our Case Study. Section 4 discusses how the use of AI-based technologies, in a context of open-data, can empower new ©The Author(s) 2018. Published by Oxford University Press. forms of diffused political participation in which citizens take more ownership of public administration. Section 5 concludes the article, describing its main contributions, limitations and possibilities for future contributions.

## 2 Setting up the discussion: AI and democracy

There are many controversies regarding the impact of democracy on development, innovation and economic development. It is commonly argued that places that are more diverse, tolerant and open to new ideas have both more knowledgeable people (Florida, 2002) and business ecosystems more prone to innovate and flourish (Nelson, 1993; Acemoglu and Robinson, 2012). Technical progress is indeed highly influenced by social institutions (Williams and Edge, 1996) and political structures (North, 1990; Rodrik, 2000). Therefore, the political configurations of a jurisdiction may alter its propensity to create and diffuse new technologies. In this article, we look at the opposite and rather neglected causal relation: technological development affecting democracy. We do this by focusing on the role AI-based technologies can play in political participation.

About 20years ago, Barber (1998: 575) raised the following question: ‘has modern technology corrupted or improved our polity?’. It seems clear that ICT technologies enabled profound societal changes and influenced the configuration of the so-called ‘knowledge societies’ (David and Foray, 2002) and ‘knowledge-based economies’ (Foray and Lundvall, 1998). These technologies eased communication, data sharing and tools for networking. More specifically, the development of AI-powered technologies exponentially augmented access to information of all sorts and created algorithms capable of processing big data quickly and efficiently. This is the case of machine learning, i.e. computer algorithms used to autonomously learn from data and information (Marr, 2016)

Studies have shown it would nonetheless be naïve to adopt a positivist approach towards technological development, one that considers them to be value-neutral and a ‘one-track race to the future’ (Savaget and Acero, 2017). Therefore, positive and negative prospects of the influence of AI on democracy enhancement and public empowerment should be further investigated. Table 1 presents two opposite scenarios, in a spectrum ranging from Pandora (in which AI only has negative effects on democracy) and Jeffersonian (in which AI only positively impacts democracy).

Deleterious effects of AI could occur, for example, if governments or powerful elites utilize new technologies for the purpose of standardization, control or repression. Algorithms are able to undermine the fairness and quality of political discourse, and they reflect the values of their designers and their intended uses (Mittelstadt, 2016). In fact, there are evidences that Bots—autonomous accounts programmed to spread messages to create the illusion of public support (Kollanyi (2016)—have been used in elections in the USA (Bessi and Ferrara, 2016; Woolley, 2016; Cadwalladr, 2017; Woolley and Guilbeault, 2017), Germany (Neudert *et al*., 2017), the UK (Howard and Kollanyi, 2016; Cadwalladr, 2017), France (Ferrara, 2017), and Brazil (Ruediger, 2017), to cite just a few. Cyber troops (Bradshaw and Howard, 2017) deploy AI-powered technologies to mislead public opinion, attempting to manipulate citizens during election campaigns by shaping public discourse and distorting political sentiment (Bradshaw and Howard, 2017; Helbing *et al*., 2017; Polonski, 2017c, 2017a, 2017b).

**Table 1:** AI-based technologies in two opposite scenarios.

	Pandora scenario	Jeffersonian scenario
Definition*	AI-based technologies can bring all evils to humanity and weaken democracy.	AI-based technologies can bring all virtues to humanity and enhance democracy.
Uses of AI-based Technologies	Facilitates centralization of control over information and communication; fake vocal political support on social media; spread false messages to create the illusion of public support; manipulate citizens during election campaigns; reinforce ‘filter bubbles’, etc.	Permit marginalized people to join the democratic process; engage voters and help them be more informed about key political issues; increase people’s voices and make sure their claims are heard by elected representatives; auditing for transparency, etc.

*Source:* Authors’ own. Note: (*) denotes definitions based on Barber (1998).

Moreover, AI-based technologies can cause ‘resonance effect’, that is, suggestions customized to each individual that are gradually reinforced by repetition and lead to ‘filter bubbles’. Such large-scale and intensive use of manipulative methods can generate social polarization, opening up scope for the brutalization of behaviour in both the digital and physical worlds, thus compromising social cohesion (Helbing *et al*., 2017).

Alternatively, on the optimistic side of the spectrum—the Jeffersonian Scenario—AI-based technologies could offer powerful assistance to democracy by bringing disenfranchised citizens closer to public administration. Political science literature recognizes that citizen participation has positive effects on the quality of democracy (Michels, 2011; Archibugi and Cellini, 2017). It would thus be desirable to open up scope for citizens to engage with political affairs, instead of relying entirely on an elite of political representatives chosen through sporadic elections (Pateman, 1970; MacPherson, 2012).

Accordingly, AI-based technologies permit marginalized people in countries with many official languages to be empowered—for instance, by providing them with automated translation tools to allow them to vote (WWWF, 2017). This is the case in India, a multilingual country with 22 official languages and many unofficial ones also in use, where AI technologies have been used to overcome language barriers (Khemani, 2012). During election campaigns, these technologies can also engage voters and help them to be more and better informed about key political issues (Polonski, 2017c), ‘overcoming regional parochialism, local prejudice and national chauvinism’ (Barber, 1998: 583) and increasing people’s voices in order to make sure their claims are heard by elected representatives (Polonski, 2017c). Furthermore, AI technologies can play an important role in extracting data from public blogs, forums and the press, as well as help policy makers outline public opinion and controversial issues, influencing policy planning and implementation (Milano *et al*., 2014).

AI-based technologies in general, and machine learning, in particular, can be major drivers pulling civil society closer to public administration by allowing citizens to tackle stable and predictable problems for which large volumes of data are relatively easy to collect. This happens through what the OECD (2016) calls ‘applied AI’: i.e. systems specialized to accomplish specific problem-solving, hypothesis-driven tasks via allowing data processing at enormous scales, hence accelerating the discovery of anomalies and patterns.

This scenario has become more promising with the sheer volume of governmental open-data associated with the proliferation of AI-based technologies and libraries (such as Google’s TensorFlow) and online repositories for open-source coding projects (such as GitHub). These have lowered barriers to entry and, consequently, widened opportunities for developers around the world to engage with public data. The open-data movement has grown considerably since the Open Government Partnership, which welcomes more than 70 countries, covers a third of the world’s population and has resulted in over 2,500 governmental commitments to disclosing public data. The idea of opening governmental data means public information should be freely available to access, use, modify and share without deliberate mechanisms of restriction or control^1^.

AI has the potential of deeply changing democracy for the better or for the worse. Similar to other radical technologies—e.g. nuclear power (Mackerron and Berkhout, 2009) and genetically modified organisms (Millstone, 2007)—the diffusion of AI-based technologies will likely become even more contentious. I other words, it can be increasingly used both with malicious or beneficial intents, and its outcomes can be differently perceived by society at large. It is thus critical to shed light over what is changing, for what reasons, by what means, and for the benefit of whom (Savaget and Acero, 2017).

Along these lines, this article focuses, specifically, on revealing how AI-based technologies can be used to empower political participation beyond elections, bringing society closer to public administration. The following section describes our research design, including how we collected, processed and analysed data in order to address our research question.

## 3 Research design

The discussion above shed light on the relationship between technological development and democratic enactment, but little is known about how emerging technologies, such as AI, can lead to changes in political regimes.

The contribution of this research thus stems from the investigation of the following question: how can AI-based technologies be used to empower political participation? We addressed this question through a stepwise, mixed-method process. We first conducted a quantitative bibliometric study, which informed a systematic literature review aimed at revealing how our topic of interest has been covered by academic publications. Based upon these results, we investigated empirical data through a qualitative and exploratory approach, which was capable of unpacking unprecedented insights addressing our research question. The following subsections present how we collected, processed and analysed our data.

### 3.1 Bibliometric analysis and systematic literature review

We conducted a bibliometric review, a meta-analytical research of literature (Kim and McMillan, 2008), to identify characteristics of articles covering AI, such as the most cited authors, keywords mentioned, the journals in which they were published and the evolution of the field over time (Bellis, 2009). This was a starting point to guide a systematic literature review. Systematic reviews, if conducted diligently, provide detailed coverage of scientific perspectives while adopting a replicable and transparent process for inclusion or exclusion of references and for analysis (Tranfield *et al*., 2003; Pittway *et al*., 2004).

Data were initially collected, processed and analysed in January 2017. However, in order to incorporate the most up-to-date references and theoretical trends, we updated our bibliometric study towards the end of November 2017. We used the search string ‘Artificial Intelligence’ to reveal all papers available on the Web of Science database, resulting in a sample of 6,773 records. These were then filtered according to academic disciplines provided by Web of Science to exclude the ones unlikely to cover topics related to our research question. The results are provided in Table 2.

**Table 2:** Overview of literature search.

Search String	Database	Filter by discipline	Results
Artificial Intelligence	Web of Science	Multidisciplinary Science	215
		Management	187
		Social Sciences Interdisciplinary	83
		History/Philosophy of Science	76
		Business	67
		Economics	62
		Social Issues	42
		Green/Sustainable Science/Technology	28
		Political Science	25
		Sociology	24
Total			727

*Source*: Authors’ own. Data sourced from Web of Science.

The 727 publications were written in seven languages: English, Spanish, French, German, Russian, Czech and Norwegian. The six papers written in Russian, Czech and Norwegian were excluded from our sample, due to our own language barriers. We then proceeded with bibliometric analysis of the remaining 721 documents. This process revealed several characteristics of the literature; among the most relevant were:

- the evolution of the field over time;
- the geographic dispersal of publications; and
- the main areas of coverage.

**Figure 1:**
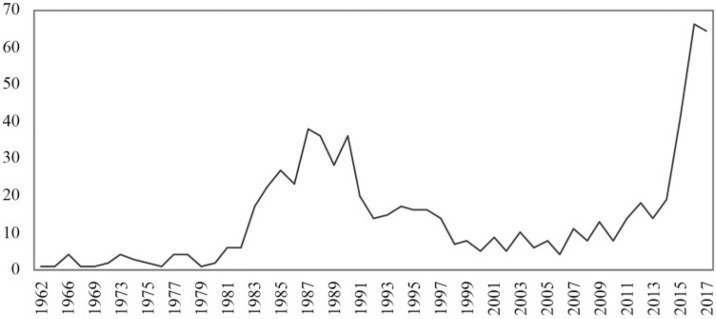
Number of publications by year. Source: Authors’ own. Data sourced from Web of Science

We used the open source software Hammer to conduct the statistical and network analysis functions (Knutas *et al*., 2015). The resulting dataset was then analysed with Microsoft Excel. Figure 1 portrays the tumultuous trajectory of the number of AI publications since the first one, dated in 1962. The number progressed slowly until the end of the 1970s, mostly due to the British and U.S. governments cutting budgets for exploratory research (Crevier, 1993). In the 1980s, AI research gained strength thanks to the commercial success of expert systems, i.e., methods that simulated the analytical skills of human experts. However, the number of AI publications fell once again into a longer hiatus, until the 2010s. Although disputed, this lapse has been attributed to the collapse of the Lisp Machine market in 1987 (McCorduck, 2004). The trend reversed abruptly in 2015. According to Clark (2015), this was a landmark year because of the rapid development and wide diffusion of AI-related technologies and techniques, such as machine learning and neural networks.

**Figure 2:**
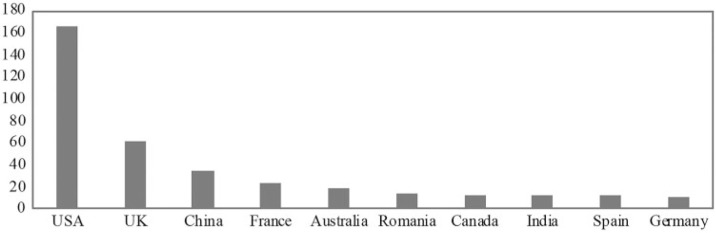
Most common geographical locations of authors. *Source:* Authors’ own. Data sourced from Web of Science.

Figure 2 shows the most common geographical location of authors. It is important to highlight that we could only track this information for a subsample of 500 of our original sample of 721. The USA presents almost three times the number of publications as does the UK, which is second in the ranking. The predominance of the USA is not surprising, since it has the most funding for AI research in the world and is the country of origin of several companies developing recent AI-based technologies, such as Amazon, Facebook, Google, and other tech giants (MGI, 2017). The UK has almost twice the number of Chinese publications on AI. The origins of AI can be traced back to Turing’s pioneering theory of computation in the UK during the Second World War, which might justify the widespread number of British publications throughout our studied timespan. The Chinese number of publications, on the other hand, is much more concentrated but has been growing rapidly since the 2010s.

**Figure 3:**
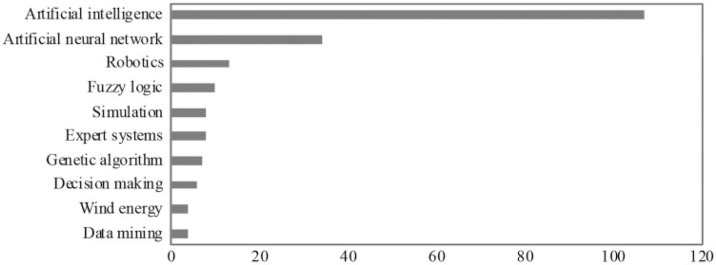
The 10 most popular keywords. *Source:* Authors’ own. Data sourced from Web of Science.

Figure 3 presents the most popular keywords. Most of them are techniques applied to AI, such as fuzzy logic, artificial neural networks, genetic algorithm, and data mining. It also presents applications of AI to enhance efficiency and optimize operations, such as for robotics and wind energy, or to assist decision-making. There are just two popular keywords closely related to politics (‘political science’ and ‘political simulation’), which belong to papers that mainly deal with how AI-based technologies can be used to aid existing governance and policymaking processes (Milano *et al.* , 2014).

After concluding the bibliometric analysis, the abstracts of all the 721 documents within our sample were thoroughly analysed to understand how the relationship between AI and political participation has been covered from 1962 (the date of the first publication on the topic) to 2017. Surprisingly, none of them mentioned political participation. The ones covering topics related to politics or policy making shied away from describing the influence of AI over democratic regimes and did not approach how civil society can use AI to take more active roles in political affairs. This observation influenced our choice for a qualitative and rather exploratory approach, which is detailed next.

### Case study methodology

The bibliometric analysis and systematic literature review revealed that the relationship between AI and political participation is a nascent domain. Our research was consequently designed according to the lack of plausible existing theory (Edmondson and McManus, 2007). This phenomenon is not only under-researched, but it is also context-dependent and open-ended. We therefore deploy inductive and qualitative methods (Gill and Johnson, 2002) to provide a deep and detailed comprehension of the investigated phenomena through a Case Study (Yin, 2008), which is particularly capable of unpacking novel insights to build theory (Eisenhardt and Graebner, 2007). The Case Study was chosen based on uniqueness and on its potential to generate novel propositions that can be further investigated, explained, and validated.

We collected data through a combination of different techniques, namely exploratory interviews, participant observation, and document analysis. Data were in both Portuguese and English and include:

- approximately 9 h of interviews with the participants of the Case Study;
- participant observation data gathered by monitoring the discussions of more than 500 collaborators through GitHub^2^ and Telegram Messenger^3^; and
- open-source materials made available by the participants involved, mostly through the online platforms GitHub and Medium^4^.

We conducted interviews via Skype in an exploratory and semistructured fashion (Robson, 2002), focusing on open-ended enquiries as well as subjective perspectives of the involved participants on motivations, prospects, and key determinants that would enable the participation of civil society in political affairs. Interviews were conducted individually with six key participants in order to prevent power plays constraining data collection, and these have been hereby anonymously identified with randomly assigned numbers (i.e. P1, P2…P6) to ensure confidentiality. Events were viewed from multiple informants’ perspectives and via complex and interconnected variables, clarified through interpretation and contextualization of their perspectives.

Participant observation was a complementary technique to the exploratory interviews, allowing the researchers to perceive reality from the viewpoint of someone observing from within rather than from an external viewpoint. It was thus possible to contrast these observations with the ones portrayed by the interviewees to best describe interactions and untold stories (Yin, 2008). We also made use of secondary materials to fill gaps when primary data did not suffice, as well as to shed light on informants’ communication with broad audiences and third parties.

After finishing data collection, we fully transcribed the interviews and analysed their content with the assistance of Nvivo^5^ software. Content analysis is recommended for analysing written communication through thematic interpretation of a given text by attentively reading documents to code relevant extracts (Weber, 1990) and, as a result, provide a condensed description of patterns. As this is exploratory research, we coded data without the support of previously established nodes and categories. Instead, these emerged during content analysis and were reframed and contrasted throughout the process. We also triangulated them between the authors of this article to minimize bias. The content analysis was combined with observations arising from both secondary materials and participant observation. We then compiled extracts for each relevant theme, unpacking novel insights that were related back to the research question and to the existing literature.

#### 3.2.1 The case

According to data sourced from the World Bank, Brazil had the ninth highest GDP in the world in 2016. In the same year, it was ranked 76^th^ in Transparency International’s Corruption Index (TI, 2017). A study done by FIESP (2010) revealed that the average annual cost of corruption in Brazil ranges from 1.4 to 2.3 percent of its GDP. If these numbers are accurate, the cost of corruption in Brazil could potentially reach up to USD 53 billion per year.

In 2011, the Brazilian government passed the Information Access Law^6^, which makes open data compulsory for all public bodies. This led to the emergence of institutional mechanisms leveraging the use of open data to encourage democratic participation and to tackle corruption. Nonetheless, open data in Brazil is still underutilized, and anticorruption enforcement is weak. Efforts to translate the increasingly available data into understandable information that can guide practical actions are still incipient (Iglesias, 2017).

While major corruption schemes are progressively under the investigation of the responsible governmental agencies, other kinds of inappropriate expenses are harder to assess and investigate, requiring human and technological efforts that go beyond the current capacity of investigative bodies. This includes the so-called Quota for Parliamentary Activity, or QPA *(Cota para o Exercicio da Atividade Parlamentar,* CEAP^7^), a fund that spends up to approximately USD 15 thousand a month^8^ to reimburse each congressperson for meals, flights, fuel, car rentals and other routine payments incurred while performing their parliamentary activities. The team responsible for receiving and processing reimbursement claims in the Lower House of the Congress receives an average of 20 thousand receipts per month. The process of checking each receipt is manual, leaving room for mistakes and corruption to pass undetected.

In 2016, a multidisciplinary group of individuals started an open and autonomous project named *‘Operaço Serenata de Amor*’ (OSA)^9^, which deploys AI-based technologies in order to empower civic auditing of public administration. These technologies were developed to analyse and report potentially inappropriate public expenses, starting with the QPA. After raising over USD 20 thousand through a crowdfunding campaign to kick-start the project, the group created an open source AI robot, known as ‘Rosie’, that uses algorithms to automatically read receipts claimed through the QPA; it then calculates the probability of irregularities and justifies its conclusions.

The deployment of AI involves a deductive, hypothesis-driven method that learns and improves itself throughout the process. OSA creates hypotheses according to the understanding of the specificities of the QPA laws and by examining the dataset, then identifying potential sources of inappropriate public expenses. Hypotheses include, to cite a few, over-invoicing, reimbursements issued by bogus companies, and expenses with products/services that are not specified (or allowed) by the law.

Thanks to AI’s continuous developments, OSA is able to gather, process and analyse an incredible amount of data that are openly available. These are used to run plausible hypotheses and find anomalies in thousands of reimbursement claims. Independently of congresspeople’s political affiliations, all anomalies are reviewed by the OSA team and reported to the responsible governmental body, following the procedure established by the Information Access Law. The responsible authority analyses each report and, if it agrees with the legitimacy of the complaint submitted, the congressperson has to justify the expense and/or give the money back.

Approximately 6 months after deploying AI to investigate the QPA, more than 8 thousand potentially irregular expenses were identified, and 629 of them—exposing 216 of the 513 congresspeople at the time—were reported to the responsible authorities.

OSA has a team of eight staff, horizontally coordinated and working from different geographical locations both within and outside Brazil, whose expenses are covered through crowdfunding. Furthermore, as the entire project is open source, OSA also works with a group of more than 500 volunteers interacting through social media, such as GitHub and Telegram, to improve Rosie’s algorithms and to assist in easing the mechanisms of reporting irregular expenses, in order to meet the bureaucratic procedure established by Brazilian law.

The algorithms and results are fully open, meaning anyone can contribute to their development, access results online, or assist with analysis and reporting. Besides the formal complaints, OSA also publicizes irregularities through online social media, such as Medium, Twitter, and Facebook (on which they have thousands of followers), allowing the media and the general public to be informed and to contact a given congressperson to ask for clarification. Over 70 media channels have already reported results from this project (P4). The OSA team plans to scale up to investigate reimbursement claims from the Brazilian Senate, public procurement of the Brazilian federal government and public administration of Brazilian cities, as well as expand to international jurisdictions that have also implemented open-data policies.

The ultimate goal is to ‘use technology to empower political change’ (P1) by promoting civic auditing of the public administration. According to P1, ‘we do not claim we are fighting corruption, which is a very broad, confrontational and imprecise term. We are assisting society to have more control of public expenses, to keep track of how public money is being used’. Besides revealing an unprecedented number of potentially irregular expenses to the responsible authorities and observing the increasing awareness and engagement of a society that has been disenfranchised from political participation beyond voting in elections, OSA measures its success by when congresspeople respond to these claims publicly and when irregular expenses are recognized and paid back. Indirect impacts include, for example, a vast number of collaborators who are learning-by-doing about AI while engaging with OSA, hence improving their professional outlooks. As OSA strictly follows an open-code policy, other groups might use their technologies and knowledge for other purposes, potentially spilling over to existing industries and indirectly assisting the generation of new endeavours.

## 4 Results and discussion

In this section, we draw from the Case Study and the literature review, firstly discussing how the use of AI-based technologies, in a context of open-data, can empower citizens to take more ownership of public administration. This is followed by the presentation of a framework to describe the focal areas, predominant traits and key features of a diffused political participation enabled by AI. We conclude this section by presenting the main challenges and limitations of AI-enabled political participation.

### 4.1 Open-data and AI

Despite being made available online, ‘datasets are often difficult to be fully digested and comprehended by the civil society’ (P3). As described by P2, ‘movements of democratic accountability and transparency revolve around making data available, but it does not mean data is being made accessible to the society at large (…). [The civil society] can and should directly benefit from the achievements of the open data movement’.

The case of Brazil demonstrates that there are powerful vested interests constraining the use of data for enforcement of anticorruption policies. It also shows institutional resistance to deploying cutting-edge technologies to process enormous datasets, which currently are often processed manually by understaffed and underbudgeted governmental agencies or non-profits.

AI thus opens up unprecedented mechanisms for civil society to process underutilized datasets and explore participatory mechanisms that influence political activities. As described by P1, ‘not only should technology be used by whom is providing information, but also by the ones who should be consuming this information’.

The case of OSA shows the pioneering use of AI-based technologies to audit public expenses. These are both conducted and funded by civil society groups in Brazil and hold possibilities of being adapted to (or even replicated in) other countries.

There are other initiatives of Brazilian civil society that do not use AI, ‘composed by experts who do everything manually, trying to identify outliers and then going, for each potential case of corruption, through dozens of websites and formal processes to obtain and contrast data. It is like finding a needle in a haystack’ (P1). Conversely, through deploying AI, efficiency grows exponentially. A civil servant responsible for auditing governmental expenses told P2 that OSA ‘in a week revealed more suspicious claims than what the responsible governmental agency did in a year’. Cases such as that of OSA are nonetheless very rare around the world. Learning from this insightful case sheds light on how civil society groups can deploy modern technologies in general and AI in particular to nurture social control of public expenses and, more broadly, to promote political participation beyond choosing political representatives in sporadic elections.

### Applied AI for diffused political participation

Based upon OSA’s case and our literature review, we summarized the key characteristics of how civil society can deploy AI to take more ownership of public administration. Table 3 describes six focal areas, their dominant traits and 23 probable features characterizing them.

**Table 3:** Key characteristics of diffused political participation enabled byAI.

Focal areas	Dominant traits	Descriptive features
Funding	Decentralized	Crowdfunding Third sector and individuals In-parallel for-profit services
Governance	Horizontal	Ethics and clear goals Organizational culture Workflow Curate and review Partnerships
Human Resources	Diverse	Multidisciplinary Sofa activism Safety net
Operations	Lean	Fill gaps ‘Small is beautiful’ System flow Pilot and experiment Immediacy, practicality and malleability
Public relations	Openness	Funding accountability Open code Legality and liability Report findings
Scaling up	Distributed	Replicability Adaptability Spill-over

*Source:* Authors’ own.

#### 4.2.1 Funding

Deploying AI to tackle governmental problems by tapping into emerging open-data contexts has low barriers to entry. However, citizen-led initiatives will still need funding both to kick-start new projects and to scale-up existing ones. Funding is needed, not only to purchase eventual technologies and licenses, but also to allow citizens—especially ICT geeks—to dedicate their time to designing and running these projects.

Funding arising directly from governments could potentially delegitimize these initiatives, even if following rigid and transparent ethical procedures. Reputational risks notwithstanding, public funding for technology-intensive services, such as AI-auditing of public expenses, are very scarce, especially in developing regions such as Brazil. Public procurement tends to be keener on focusing exclusively on very mature technologies and to be influenced by vested interests, which are opposed to the very purpose of these initiatives. For these reasons, initiatives such as OSA are prone to be funded in a very decentralized fashion, mostly by civil society itself.

Crowdfunding has proven to be particularly good at kick-starting technology-intensive projects, and, in this case, it also empowers civil society to participate and to join the project, building up momentum for positive and collective change. According to P2, not only was crowdfunding very helpful for funding, but also ‘to push our initiative to create and to test a concept’, similar to the creation of an open and easily comprehended business plan that would be presented to potential funders. P1 emphasized that crowdfunding ‘promotes autonomy, both to people who are leading the project and to the ones supporting the project’. At the same time, it nurtures ‘micro-communities and it deals better with the lack of trust of the civil society’ (P1) towards governments and large organizations.

After kick-starting, these initiatives can use recurring crowdfunding, in which donors commit a certain monthly amount to the project. P3 emphasized that this posits a pressure to keep up, to perform well and to constantly present results. However, this source of funding is unlikely to meet all expenses, especially when incrementally expanding the initiative towards new jurisdictions or adding new functionalities. Donations and grants from third sector organizations, such as private foundations, or wealthy individuals can fill this gap, but these are unlikely to be obtained to kick-start novel projects led by diffused civil society groups. Once the results are unpacked and the initiative gains more support and visibility, it becomes better positioned to apply for grants and donations. Similar to crowdfunding, grants often come with strings attached. On one hand, a grant does not provide permanent stability to the initiative and financial security to its most engaged members, but it sets models in the form of discrete projects, from design to implementation of these initiatives, with the potential to be replicated or adapted elsewhere.

Financial security is indeed an important matter for the development of these initiatives. P2 declared, ‘we cannot work full-time for free (…) but we cannot let OSA die either’. This idea was endorsed by all other interviewees. The case shows that a possible solution is in-parallel for-profit services to clients demanding AI solutions. The core team created a company, hoping to raise enough revenue streams through in-parallel services to sponsor most of their expenses with OSA. The latter would still be kept as an open-source, not-for-profit project. It is interesting to observe that, after starting OSA, the core members not only benefitted from it by using the project as a ‘lab’ to experiment and develop their AI skills, but also improved the outlook of their resumes and gained credibility enough to start a company with a proven track record and portfolio.

#### 4.2.2 Governance

Horizontal governance is not only desirable, but also a matter of analytical rigor, if dealing with open-source initiatives to empower civil society. Horizontality does not mean a lack of goals and formal ethical standards. The OSA case has shown, for example, that, when integrating a wide range of collaborators, the initiative might risk shying away from its main targets or adopting a rather aggressive, confrontational approach that could undermine the credibility of the project (P1, P2, P4).

Ethical standards and goals need to be explicit and shared among all collaborators. In the case of OSA, it was critical to emphasize that the initiative does not aim at fighting corruption, but rather at empowering civil society to take more ownership of public administration. Otherwise, as described by P1, ‘a collaborator can hack private details of a potentially corrupt politician, such as his address, and start sending pizzas to his house. However, this is not what we want to do (…). We do not want to make the lives of politicians a hell and shame them in public arenas’.

The OSA case also demonstrates the importance of ensuring an organizational culture based on trust, as well as a tolerant, diverse and collaborative organizational culture. P5, for example, emphasized that she does not ‘like working in environments where I feel affected for being a woman, and this happens a lot in technology (…). I could be doing millions of other things, working for a large company, but they are often misogynist, and I feel comfortable and respected working at OSA’. P6 described that ‘social control of the government was never something very dear to my heart (…). I wanted to work with data analysis, and the team members know a lot. Working with governmental data came as a cherry on the top of the cake. My main incentive though is learning’.

One of the most challenging features of horizontal governance, with the team performing fluid roles and working remotely and flexibly, is managing workflow. The OSA case has shown the importance of maintaining stable communication. According to P6, ‘we have daily meetings, at 9 am, lasting 15 min each. The idea is to present what you did the day before, what you will do today, and to tell if you need something (…). With this brief communication, we know what is being done, by whom, and when it will be delivered’. As emphasized by P5, in these meetings, ‘we do not have to ask for permission, the main concern is not if I *can* do something, but rather if I *should* do it’. They also use two other techniques, well-known among programmers. One is called time-boxing, in which a fixed maximum time period, or ‘time box’, is allocated to each planned activity, and, ‘if that time was not enough, you skip that task and still go to the following one’ (P5). The other consists of remote pairing, in which two programmers in different locations work together on a task, using tools such as a collaborative real-time editor, shared desktop and time markers—’not to tell how many hours per day you have to work, but rather to show your availability to perform that task’ (P5). Besides improving team satisfaction, knowledge sharing and reduction of code defects, pair programming is particularly advantageous for exploratory tasks involving emerging technologies, such as AI, in which programmers might not know beforehand what the process will entail (Cockburn and Williams, 2001; Lui and Chan, 2006).

It is also critical to ensure a process of reviewing codes and curating analytical content, especially from new or infrequent collaborators. P1 described that OSA has an implemented system of code review, in which ‘we read the code from all collaborators, to identify if there is a loophole, if it makes sense mathematically, and if it is coherent with the hypothesis (…) as there are barriers that are technological, but also ones that are related to legal knowledge’. It is also important to curate analytical content. P1 illustrated with a case in which ‘I gave a feedback to an analysis that used the language of ‘criminal’ to refer to the politician. Nevertheless, we are only dealing with suspicious things, we are not the judicial system. Then I explained we are talking about statistics, probabilities, hence we need to use the language of suspicious expenses’.

Partnerships are very important. In the beginning, they tend to happen mostly with given individuals acting as mentors who are willing to share knowledge and expertise on critical and complementary topics. OSA consulted with three individual mentors with very different expertise when kick-starting: a specialist in open-data and data science, a lawyer who helps identify legal processes and liabilities of OSA’s operations and another who helps with fundraising. After kick-starting, the project is better positioned to interact with mainstream agents, including members of non-profits working towards similar purposes and employees of governmental bodies auditing public expenses. These partners are very important and ‘helped identifying the best pathways to pursue, where the bottlenecks are’ (P2). However, especially within the government, ‘some of them might not want to identify themselves’ (P3) in order to avoid political clashes and retaliation. Therefore, support tends to be individualized rather than institutionalized.

#### 4.2.3 Human resources

Empowering civil society through AI-based technologies also implies cultivating a diverse pool of human resources. This allows the team to redefine problems outside rigid disciplinary boundaries and focus on finding complementarities. As a result, it improves the likelihood of coping with changing political scenarios, deploying emerging and uncertain technologies and approaching complex multi-stakeholder situations. Although the majority of team members have programming knowledge, the OSA case clearly shows how they deliberately involved other, complementary skills, such as administrative, journalistic, and legal ones. Besides better end results, P5 emphasized better internal and external communication—for example, as laws ‘are difficult to understand, written by lawyers and for lawyers, we created a simple version, that anyone can understand, by summarizing what can be done and what cannot’. P6 also described ‘we are constantly learning from others and, as we recognize skills and appreciate different inputs, decision-making happens without conflicts’.

The citizens programming AI algorithms are likely to be from the so-called millennial generation, which is marked by being very urban, having an increased familiarity with digital technologies and presenting a more liberal approach towards politics. This generation is also more prone to adopt a ‘sofa activist approach’ towards political engagement. Critics have characterized this behaviour by labelling them ‘slacktivist’. UNAIDS (2010: 143), for example, described slacktivists as people who support causes by performing simple, ‘feel-good’ measures instead of being ‘truly engaged or devoted to making a change’. However, as highlighted by P1, ‘sofa activism might not be as useless as it seems (…). OSA was done entirely by people on different sofas. I was programming while sitting on my sofa with my dog underneath my feet’.

In order to leverage this generational and rather international culture, it seems critical to use open-source and open-code tools, as well as to develop codes and conduct most technical communication in English. As emphasized by P4, ‘we had to opt between a few people who know how to program but do not speak English, or people in the entire world seeing and potentially collaborating’. It is also very important to manage these communities. As described by P1, ‘we give them an explanatory map, like the one you receive when you go to the museum with the galleries (…) and show what we understand as good practices’. P5 emphasized the need for ‘non-violent communication’, since ‘we are dealing with an open group [for political engagement], and we will not expel anyone’. Coding sprints have also proven, according to P6, to be very beneficial in building up momentum and engaging people who cannot steadily work on the project. These sprints consist of getting developers to work on a given project for a set period of time, often a weekend.

Furthermore, consistent with literature on entrepreneurship [e.g. Blanchflower and Oswald (1998)], the participants, especially the ones who are fully dedicated to kick-starting these projects, are likely to have a safety net. When basic needs are met, such as housing and food, it is easier to be creative, to take risks and to renounce stable jobs in order to pursue more pleasurable professional options. In the beginning, OSA was led by people who were more affluent than the population’s average and who knew, that if OSA did not succeed, they would not face socioeconomic deprivation. As highlighted by P1 and P2, OSA started as a project they conducted in their free time, and then they decided to take the risk and dedicate themselves fully to making it work. P6 accordingly said that ‘OSA is something I will work on, earning money or not. If it ends, I’d eventually have to search for something else, but OSA will still be part of my daily routine, because I do it for pleasure’.

#### 4.2.4 Operations

Initiatives to empower political participation through AI might be financially constrained and count on little institutional support before kick-starting. The team is scattered among multiple geographical locations, contributions are fully transparent, and barriers to entry and to scaling up these initiatives to other jurisdictions are low. For these reasons, operations focus on filling existing gaps, or finding the highest returns for minimal effort. OSA, for example, identified that the responsible auditing authorities are already putting great effort into revealing grand corruption schemes, such as the ones involving public procurement. According to P1, these agencies are ‘doing it well, and we need to understand that to avoid overlaps and time loss’. However, they cannot investigate suspicious expenses that are relatively small, as the amount of investigations would be too high to be done manually. P2 described being told by an employee of an auditing agency that ‘expenses lower than approximately USD 25 thousand cannot be properly investigated. If agencies look at big, we [OSA] can look at small, and then citizens will not have blind spots’. AI-based technologies could thus fill this gap while concomitantly building up momentum to mobilize civil society to take more active political roles.

The expression ‘small is beautiful’ was coined by Schumacher (1973) and shed light on how technologies should be appropriated to empower people. We are hereby paraphrasing Schumacher (1973) to describe how AI-based technologies can be best used to empower citizens to participate in politics. At least in the initial stages, it seems that AI should be people-centred and used to fill gaps, such as small irregular expenses that pass unnoticed by auditing agencies but whose data is openly available. As described by P2, ‘we want to put the citizens in the conversation (…), to debate the use of public money. Not only the bombastic political news… We want them to think about the day-to-day of politics, the expenses happening on Monday, to let them interact directly with the politician’.

It also seems critical to tap into potential leverage points of the system, i.e., places within complex systems where a small shift in one thing can produce big changes in everything (Meadows, 2009). OSA identified, for example, that, while highlighting individual irregular expenses signals potential wrongdoings of politicians and mobilizes routine political participation, these spotlights have little potential to cause short-term stress in the dominant political system. By grouping thousands of inappropriate expenses together, OSA could then amplify its capacity to advocate for changes in the legislation, so as to eliminate what they consider to be spurious or unnecessary public benefits to politicians on the grounds ‘that they have been systematically misused’ (P1).

Similar to the design and implementation process of business model innovations, pioneering initiatives employing AI to empower political participation are more likely to succeed if they prototype, pilot and experiment. It is particularly critical to learn, test and validate concepts and variables in early stages, as well as to rollout endeavours (Chesbrough, 2010). As described by P2, OSA ‘did a pre-analysis of the data even before launching the crowdfunding to be sure ‘there were wrong things there that can be found’. Then the first round of crowdfunding was mostly to develop the concept even further and to validate our assumption that a robot could audit public expenses’. This shares similarities with an established business model literature that has seen exponential growth via the wide diffusion of the concept of ‘Lean Startup’ (Ries, 2011).

In a time when information is increasingly more diffused, decentralized, and fast-paced, technology-intensive operations promoting transparency, accountability, and political participation should aspire to immediacy and practicality. As described by P1, ‘there are lots of complex theoretical criticism to representative democracy, but in practice change happens only by trial-and-error, experimenting to see what is effective and what is not’. Practicality is also evident in the choice of the QPA to kick-start the project: ‘we could start with it, because the database was very organized and very transparent’ (P4). OSA’s immediacy derives from the fact that it is a financially unstable initiative and mostly composed of millennials, who are more likely to commit to pursuing short-term outcomes: ‘we had to promise something that people would see results in as little as 2 months’ (P2).

#### 4.2.5 Public relations

The term ‘public relations’ is often used to describe a means of establishing and maintaining connections with target audiences. However, in the case of initiatives empowering political participation, the public is not merely an audience. Instead of only reporting to them, these initiatives should aim at constantly integrating civil society into their processes. The OSA case has shown how openness and transparency permeate their relationships with different civil society groups. Their bookkeeping is openly available to society at large, and most especially those who donated through crowdfunding.

A very important characteristic of OSA is that all their codes are openly available on GitHub to potential collaborators or to those who want to verify the results they find. OSA not only taps into a vast pool of collaborators to develop their AI, but also, by keeping their initiatives transparent, they are better shielded from criticism. As described by P1 ‘if we did not have open-code, we could easily be labelled and delegitimized as leftist, or rightist, or serving interests of conspirators’.

Following legal procedures is essential to avoid liabilities that can undermine or even obliterate initiatives empowering political participation, possibly directly compromising the lives of those actively involved. The OSA case demonstrates that they have to be careful with how results are reported and the language used in order to, for example, avoid legal suits of defamation after publicly exposing politicians’ names. To circumvent this liability, OSA first reports suspicious expenses to public authorities; then, after receiving their responses, the information can be fully disseminated, as the team’s work is backed up by an official public response.

Besides anticipating liabilities, initiatives should aim to expand the reach of their reporting and to expand scope for direct involvement of citizens. OSA reports findings through online social networks, such as Facebook and Twitter, and often gets coverage by the media. The former has been progressively more important in allowing civil society to engage with everyday politics: for example, by contacting politicians to request justifications. Outreach through media is also relevant, since traditional broadcasting has more outreach to those excluded from online social networks (i.e., the elderly, members of lower social strata and residents of remote geographical locations). P4 emphasized their ‘commitment with ensuring the media will always publish the entire data’. In other words, OSA only assists media channels if they commit to publishing the data as they were received, thus avoiding the use of OSA’s results to privilege vested political interests of media corporations.

#### 4.2.6 Scaling up

When initiatives empowering political participation through AI are open-code and fully transparent, there is no ownership involved, and, consequently, possibilities of scaling up become fundamentally distributed. Expansion is difficult to track, since initiatives do not need to directly involve the original team: anyone, in principle, can build upon openly available contributions. In the case of scaling up OSA’s experiences, for example, virtually every public expense can be audited by civil society, so long as there is a governmental open-data policy. As phenomena like OSA are still rare, little is known about how scaling up these initiatives can occur. However, the OSA team has identified at least three potential pathways: replicating, adapting, or spilling-over.

OSA can be replicated, for example, by using AI in similar contexts and with the same purposes. An example is scaling up coverage from expenses of politicians in the lower house to include those of the upper house of the congress. Scope here lies mostly in applying the knowledge and the robots to a similar scenario that has not yet been contemplated.

Adaptation happens when contexts are different or if the purpose changes. OSA has ambitious aims of, for example, expanding beyond Brazil to impact ‘a billion people in 10 years’ (P2). The purpose would still be the same, one of auditing public expenses to empower political participation. On one hand, they would need to understand specific, contextual characteristics and nuances, since needs, legislation and socio-political behaviours vary across jurisdictions. On the other hand, their AI solutions and a growing international community of programmers already mobilized through open-code platforms can be used as a starting point. Likewise, if a new community, independent from OSA, aims to apply AI, either in Brazil or elsewhere, with the goal of promoting political empowerment through approaches other than via auditing public expenses, it can also adapt OSA’s approach by building upon their materials, codes, and knowledge.

Finally, the OSA team has identified that the project has a great capacity as regards spilling-over practices of governments and companies alike. Governmental agencies, for instance, can start deploying AI internally to audit public expenses and enhance their investigative capacity. As highlighted by P2, robots are more efficient than people at performing a variety of tasks, and there should be room in governments ‘to allow robots to replace some of their established processes, so long as the codes are open and fully transparent’. Companies can also learn from OSA’s experiences to develop AI technologies with a wide variety of profitable purposes, such as marketing new solutions, optimizing operations, and reporting more transparently to shareholders and stakeholders.

## 5 Final remarks

We cannot fully predict whether the development of AI-based technologies will radically change the existing political paradigm, easing a shift towards a Direct Democracy or other unforeseen form of political participation. Neither can we divine what the potentially deleterious impacts of their diffusion might be. However, it seems clear that AI developments can and should be deployed to enable more diffused forms of political participation, empowering citizens to take more ownership of public administration.

The emerging context of governmental open-data and online open-source repositories for coding projects are likely suited to enable the proliferation of AI-based technologies for political empowerment. Together, they can enable civil society to participate in political affairs without requiring physical groupings of individuals. Citizens can thus become more politically active than by merely choosing representatives, instead monitoring activity and pressing for desired changes in public administration.

Our multi-method approach revealed that the literature is still very nascent. A sample of 721 publications was covered, first through a bibliometric analysis that was particularly focused on the evolution of the field over time, the geographic dispersal of publications and the main areas of coverage. This was followed by systematically reviewing the entire documents of the articles that mentioned politics in their keywords, and the abstracts of the remainder. It became clear that nothing has been academically reported on how society can use AI to promote political participation.

We then proceeded to analyse an insightful case through an exploratory, qualitative approach. This stage revealed a framework for how civil society can use AI to nurture diffused political participation. Its components arose from the experiences and expertise of those involved with OSA, crosschecked and enriched by the literature on system change, innovation, and political science summarized in Section 2. This framework consists of six focal areas: namely, funding, governance, human resources, operations, public relations, and scaling-up. Their respective dominant traits and descriptive features were scrutinized and discussed, aiming at contributing to guiding academic studies, as well as civil society endeavours.

Finally, the limitations of this work derive from the methodological approaches employed. The systematic review of academic literature exclusively used the database Web of Science for data collection. Consequently, potentially interesting articles might not have been included in our sample. At this stage, we also ignored non-academic articles, which can be particularly insightful for emergent topics. Our qualitative approach, on the other hand, was very exploratory and epistemologically inductive. That means we focused on exploring the whole of the case—instead of validating or testing its parts for generalizability—and our sample was thus small, but analysed in depth (Yin, 2008).

Besides building upon our research to validate our findings, there is a wide range of opportunities for future research in this area. Of these, we believe the following should be encouraged as being particularly critical to the progress of literature and practice:

- What changes is AI likely to cause for public bureaucracies, including (but not restricted to) job losses?
- What are the main challenges and bottlenecks constraining civil society from organizing itself to deploy AI for political participation?
- What forms of political participation are best with AI? What forms are not well-suited?
- Does civil society desire all kinds of political participation?
- What are the impacts of AI-based technologies on secrecy and national security?

## Notes

1. http://opendefinition.org/licenses/

2. https://github.com/

3. https://telegram.org/

4. https://medium.com/

5. https://www.qsrinternational.com/

6. Law no. 12,527/2011. http://www.planalto.gov.br/ccivil_03Z_ato2011-2014/2011/lei/l12527.htm

7. http://www2.camara.leg.br/legin/int/atomes/2009/atodamesa-43-21-maio-2009-588364-norma-cd-mesa.html

8. http://www2.camara.leg.br/legin/int/atomes/2015/atodamesa-4-25-fevereiro-2015-780188-publicacaooriginal-146197-cd-mesa.html

9. https://serenatadeamor.org/
